# Supplementary material for: The interhemispheric CA1 circuit governs rapid generalisation but not fear memory
Source: Nat Commun. 2017 Dec 19;8:2190. doi: 10.1038/s41467-017-02315-4 (PMC5736595; doi:10.1038/s41467-017-02315-4)
Supplement: Supplementary file 1 — Supplementary Figures [file 41467_2017_2315_MOESM1_ESM.pdf]

The interhemispheric CA1 circuit governs rapid generalisation but not fear memory

Heng Zhou[1,2#], Gui-Jing Xiong[1#], Liang Jing[1,3#], Ning-Ning Song[4#], De-Lin Pu[1], Xun Tang[2], Xiao-Bing He[5], Fu-Qiang Xu[5,6], Jing-Fei Huang[7], Ling-Jiang Li[8], Gal Richter-Levin[3], Rong-Rong Mao[1], Qi-Xin Zhou[1*], Yu-Qiang Ding[4,8*], Lin Xu[1,2,6,8,9*]

[1]Key Laboratory of Animal Models and Human Disease Mechanisms, Kunming Institute of Zoology, Chinese Academy of Sciences, Kunming 650223, China.

[2]School of Life Sciences, University of Science and Technology of China, Hefei 230027, China

[3]Department of Neurobiology and Ethology, and Department of Psychology, University of Haifa, Haifa 3498838, Israel.

[4]Key Laboratory of Arrhythmias, Ministry of Education, East Hospital, and Department of Anatomy and Neurobiology, Collaborative Innovation Centre for Brain Science, Tongji University School of Medicine, Shanghai 200092, China. Current address: Institute of Brain Sciences, Fudan University, Shanghai 200031, China

[5]Wuhan Institute of Physics and Mathematics, Chinese Academy of Sciences, Wuhan 430071, China.

[6]CAS Centre for Excellence in Brain Science and Intelligent Technology, Shanghai 200031, China.

[7]State Key Laboratory of Genetic Resources and Evolution, Kunming Institute of Zoology, Chinese Academy of Sciences, Kunming 650223, China.

[8]Mental Health Institute, Second Xiangya Hospital of Central South University, Changsha 410011, China.

[9]KIZ-SU Joint Laboratory of Animal Model and Drug Development, College of Pharmaceutical Sciences, Soochow University, Suzhou 215123, China.

#These authors contribute to this work equally.

*Correspondence: qixin_zhou@vip.126.com or dingyuqiang@vip.163.com or lxu@vip.163.com.

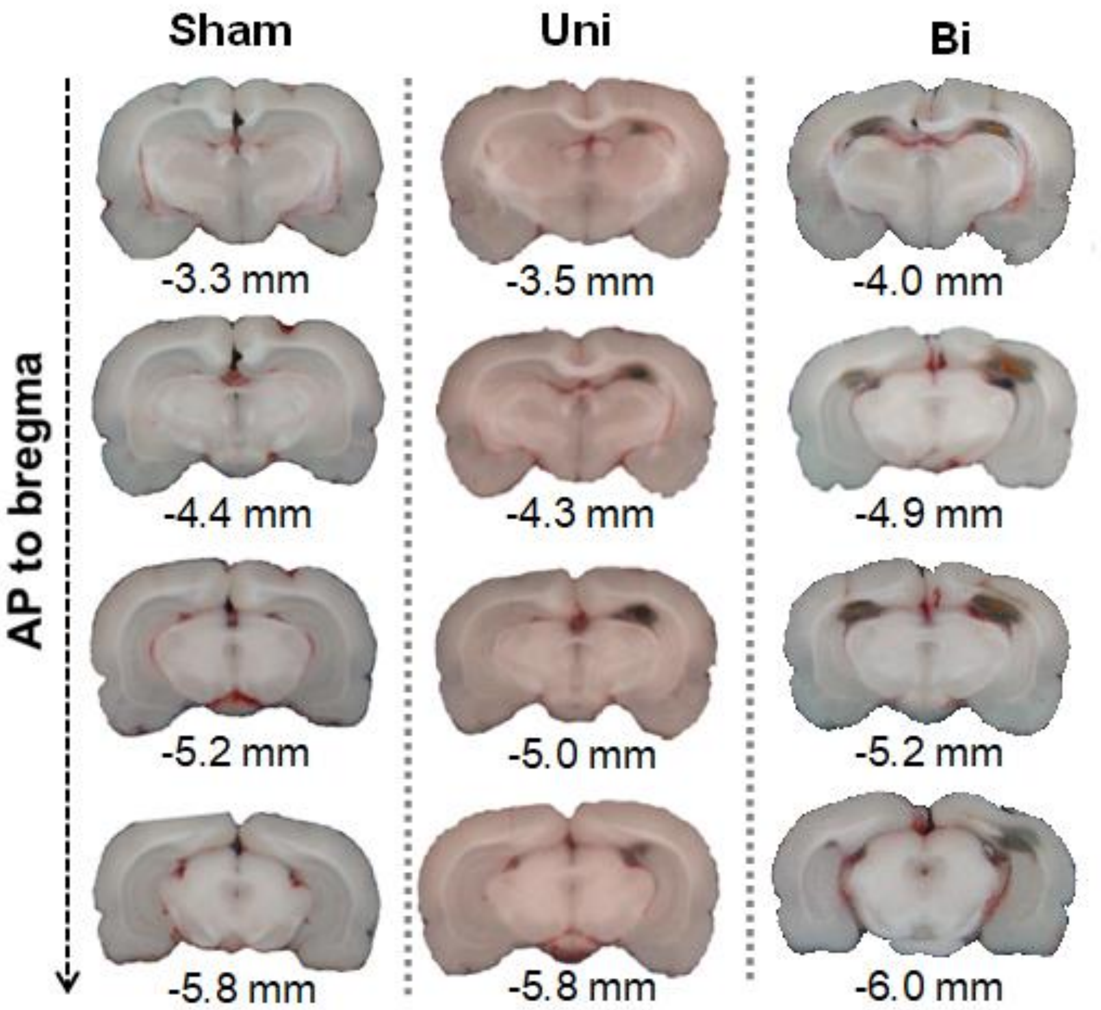

Supplementary Fig. 1 | Representative of coronal slices anterior-posterior (AP) from bregma for the CA1 lesion sites after behavioural studies. Sham, experienced all of the surgery procedures except ibotenic acid injection. Uni, unilateral CA1 lesion, and Bi, bilateral CA1 lesion, by using ibotenic acid 25 d before fear conditioning.

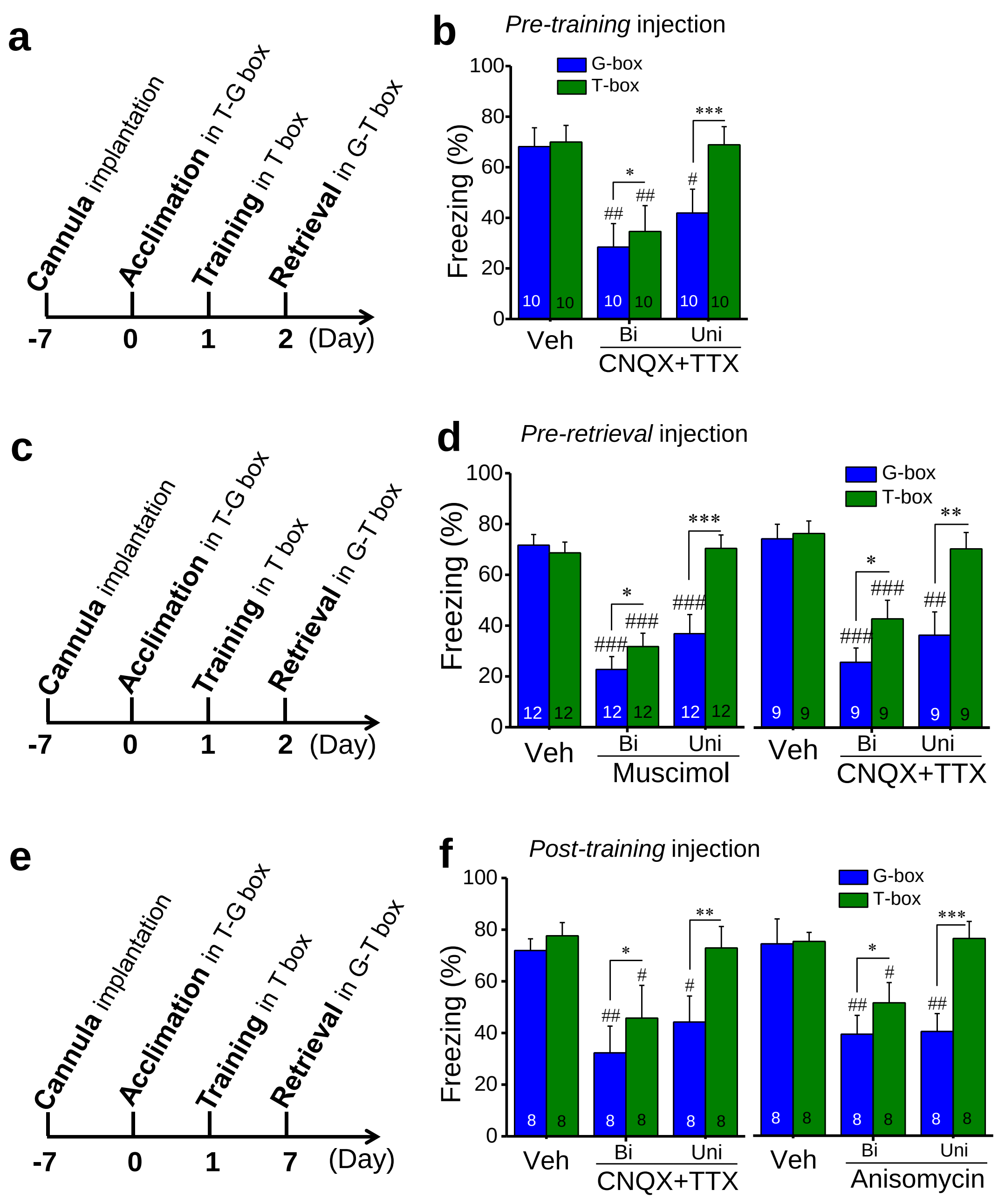

Supplementary Fig. 2 | Bilateral CA1 were needed for generalization. a, **b**, CNQX+TTX was infused into unilateral (Uni) or bilateral (Bi) CA1 before fear conditioning through implanted guide cannulas. Uni CA1 infusion impaired generalization [Group×box interaction, F (2,27) = 22.727, *P* < 0.001; Uni, #*P* < 0.043, G-box *vs.* Veh; ****P* < 0.001, G-box *vs.* T-box; two-way ANOVA] with no effect on fear memory (Uni, *P* = 0.925, T-box *vs.* Veh), but Bi CA1 infusion impaired both fear memory and generalization (Bi, ##*P* = 0.005 or 0.003, T-box or G-box *vs.* Veh; **P* = 0.02, T-box *vs.* G-box), examined 24 h after fear conditioning. **c**, **d**, Muscimol or CNQX+TTX was infused into Uni or Bi CA1 before retrieval tests. Similarly, Uni CA1 inhibition impaired generalization (Group×box interaction, F (2,33) = 11.047, *P* < 0.001; Uni: Muscimol, ###*P* < 0.001, G-box *vs.* Veh; ****P* < 0.001, G-box *vs.* T-box; Group×box interaction, F (2,24) = 10.724, *P* < 0.001; CNQX+TTX, ##*P* = 0.001, G-box *vs.* Veh; ***P* = 0.001, G-box *vs.* T-box; two-way ANOVA), but Bi CA1

inhibition impaired the both (Bi: Muscimol, ###$P < 0.001$, T-box or G-box *vs.* Veh; *$P = 0.02$, G-box *vs.* T-box; CNQX+TTX, ###$P < 0.001$, T-box or G-box *vs.* Veh; *$P = 0.011$, G-box *vs.* T-box), examined 24 h after fear conditioning. **e**, **f**, Uni or Bi CA1 infusion of either CNQX+TTX or anisomycin immediately after fear conditioning impaired generalization (Group×box interaction, $F (2,21) = 8.578$, $P = 0.006$; Uni: CNQX+TTX, #$P = 0.036$, G-box *vs.* Veh; **$P = 0.002$, G-box *vs.* T-box; Anisomycin, Group×box interaction, $F (2,21) = 26.023$, $P < 0.001$; ##$P = 0.001$, G-box *vs.* Veh; ***$P < 0.001$, G-box *vs.* T-box; two-way ANOVA) or the both (Bi: CNQX+TTX, #$P = 0.024$ or ##$P = 0.004$, T-box or G-box *vs.* Veh; *$P < 0.02$, G-box *vs.* T-box; Anisomycin, #$P = 0.019$ or ##$P = 0.001$, T-box or G-box *vs.* Veh; *$P = 0.015$, G-box *vs.* T-box), examined 7 d after fear conditioning. Statistical comparisons are performed by using #parameter estimates and *contrast effects of two-way ANOVA. # or *$P < 0.05$, ## or **$P < 0.01$, ### or ***$P < 0.001$. T-box, retrieval at the training box; G-box, retrieval at a non-training similar box; Veh, vehicle. Error bars, s.e.m.

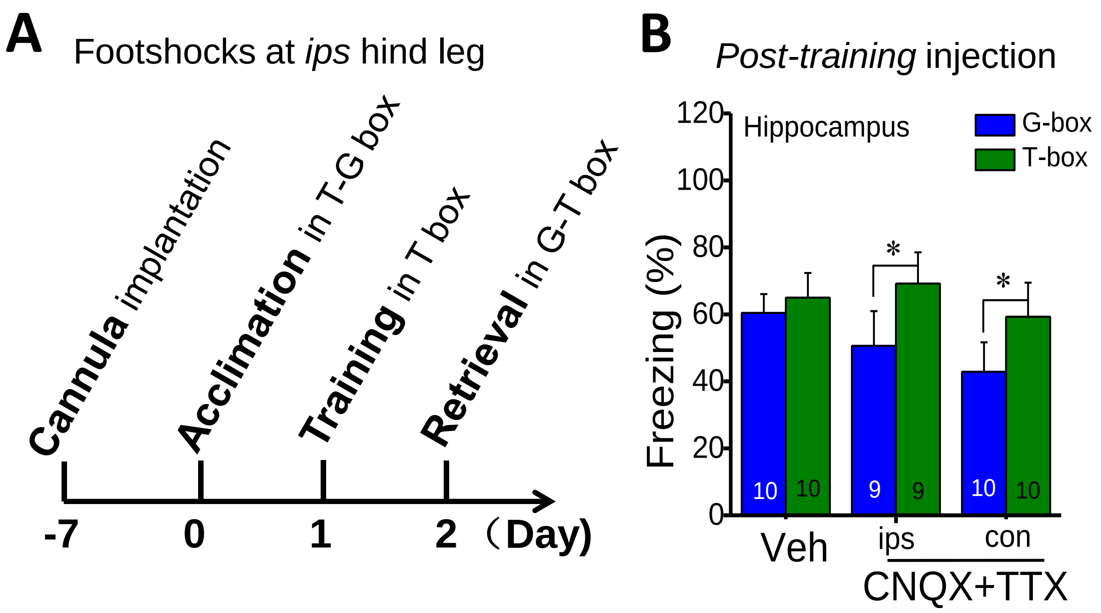

Supplementary Fig. 3 | Bilateral CA1 were required symmetrically for generalization in a single-foot fear conditioning. a, **b**, Near identical results, impaired generalization, were found by ipsilateral (ips) or contralateral (con) CA1 infusion of CNQX+TTX after fear conditioning (Laterality×box interaction, F (1,26) = 1.488, *P* = 0.224; ips, **P* = 0.02; con, **P* = 0.046; T-box *vs.* G-box; two-way ANOVA), relative to the footshocks applied at *ips* (right) hind leg of the rats. Statistical comparisons are performed by using two-way ANOVA (*contrast effects for G-box *vs.* T-box). **P* < 0.05. T-box, retrieval at the training box; G-box, retrieval at a non-training similar box; Veh, vehicle. Error bars, s.e.m.

RV-dG-Dsred

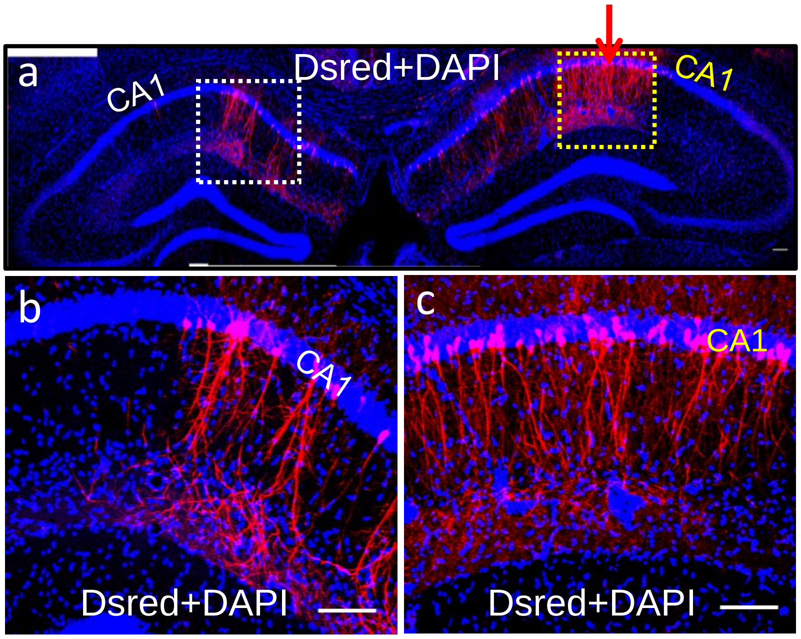

Supplementary Fig. 4 | The direct ipsCA1-conCA1 connections in the mouse. a, Non-trans-synaptic rabies virus (RV-dG-Dsred) was injected (arrow) into the stratum oriens of right CA1, and neurons were labelled in left CA1, indicating that left CA1 had direct projections onto right CA1. **b**, Magnification of the white square in left CA1. **c**, Magnification of the yellow square in right CA1. ips, ipsilateral; con, contralateral; Dsred, red; DAPI, blue. Calibration bar: 100 µm.

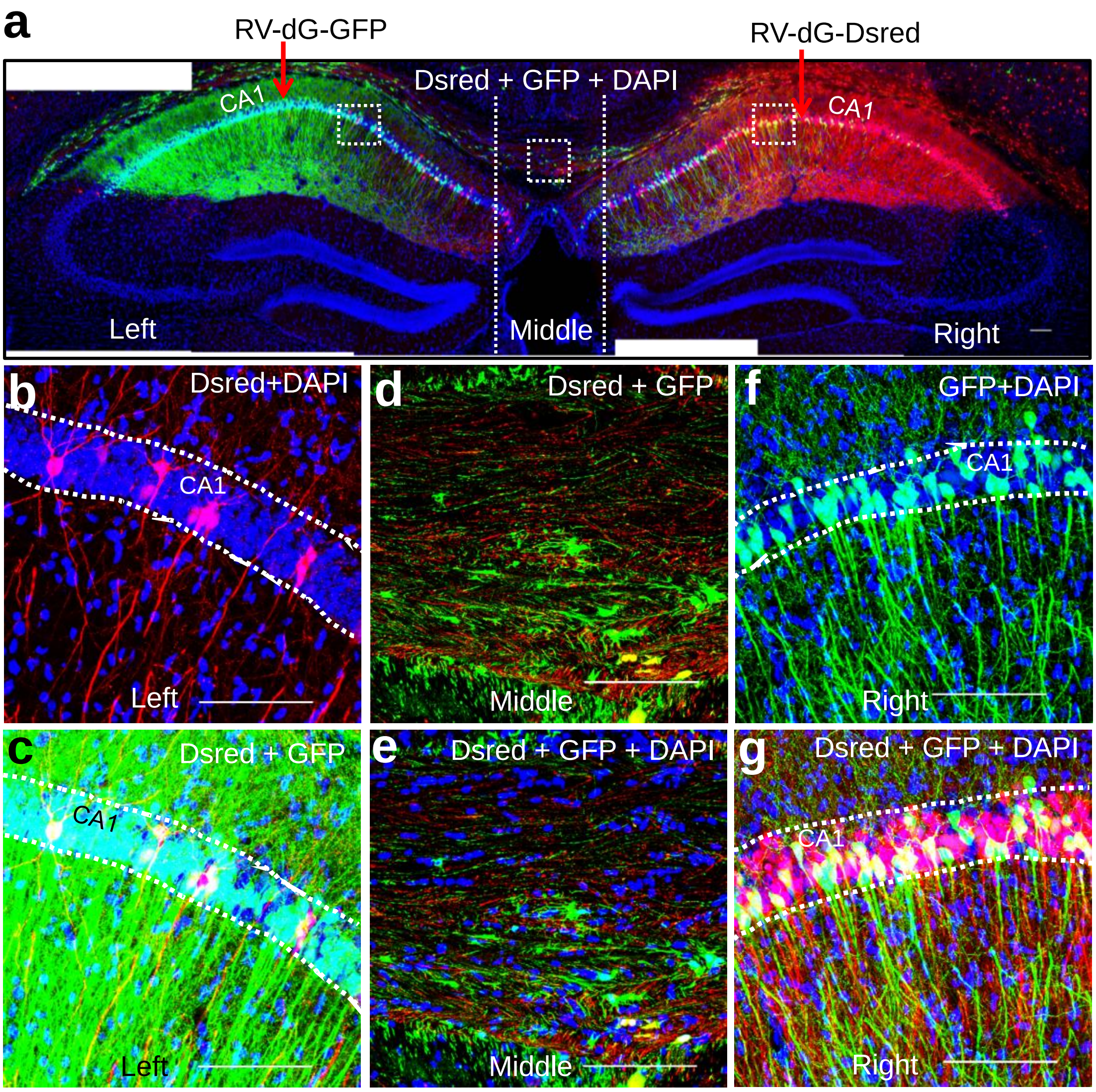

Supplementary Fig. 5 | The direct and reciprocal ipsCA1-conCA1 projections in the mouse. Non-trans-synaptic rabies virus (RV-dG) was used to trace the interhemispheric CA1-CA1 projections in the mouse. **a**, RV-dG-GFP and -Dsred were injected into left and right CA1 regions, respectively, and tens of neurons were labelled in the opposite side, demonstrating direct monosynaptic projections. **b**, **c**, left side; **d**, **e**, the dorsal hippocampal commissure (DHC) below the corpus callosum; **f**, **g**, right side. ips, ipsilateral; con, contralateral; GFP, green; Dsred, red; DAPI, blue. Calibration bar: 100 µm.

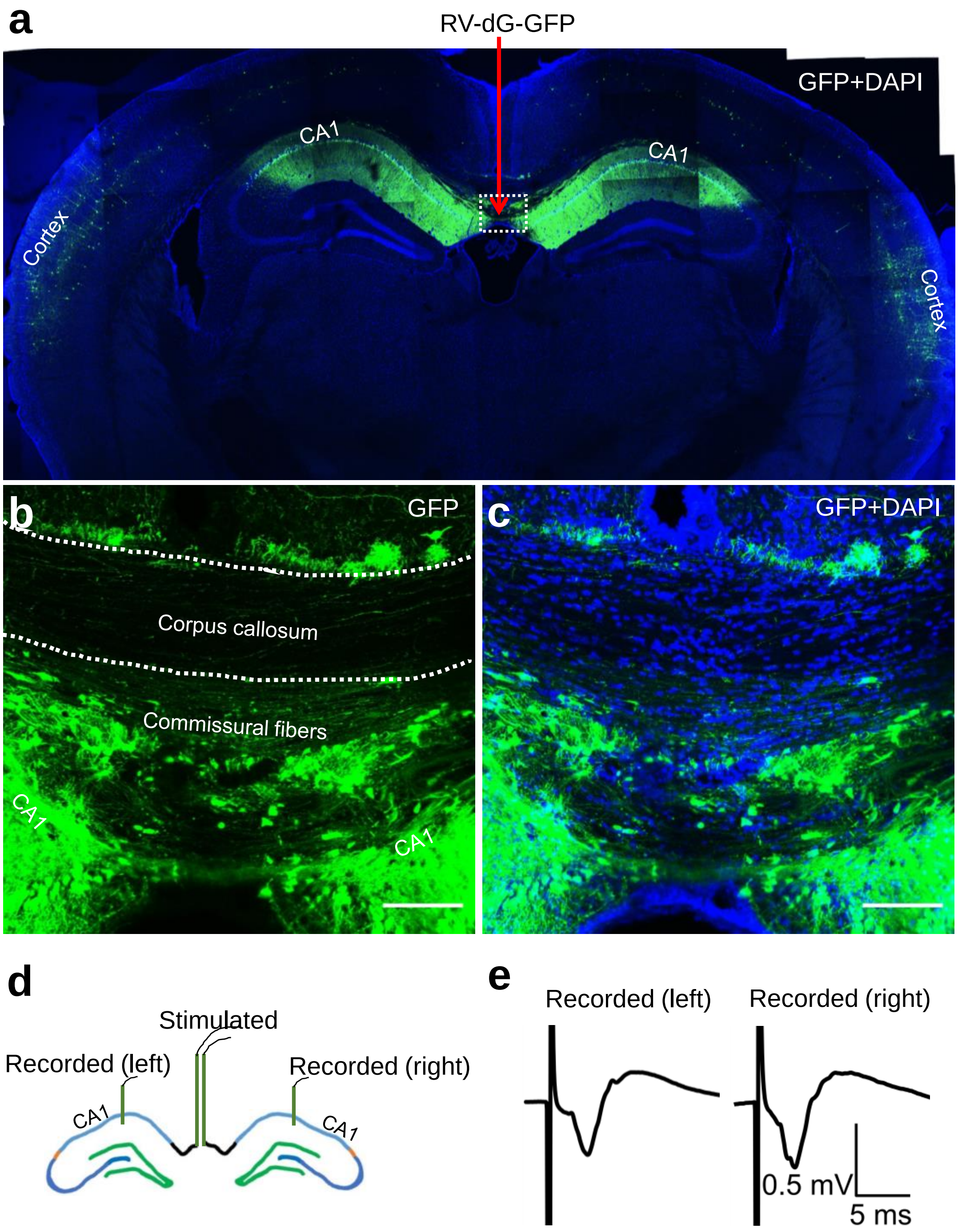

Supplementary Fig. 6 | Infusion of RV-dG-GFP into the midline below the corpus callosum in the mouse. **a**, RV-dG–GFP virus (non-trans-synaptic tracing) was injected into the midline as indicated by the arrow (red), and the whole bilateral CA1 regions were exclusively labelled in the hippocampus. **b**, **c**, The midline below the corpus callosum. **d**, **e**, In coronal slice from the mouse, stimulating at the midline below the corpus callosum effectively evoked the fEPSP in both left and right CA1 regions, suggesting direct functional connectivity. GFP, green, DAPI, blue. Calibration bar: 100 μm.

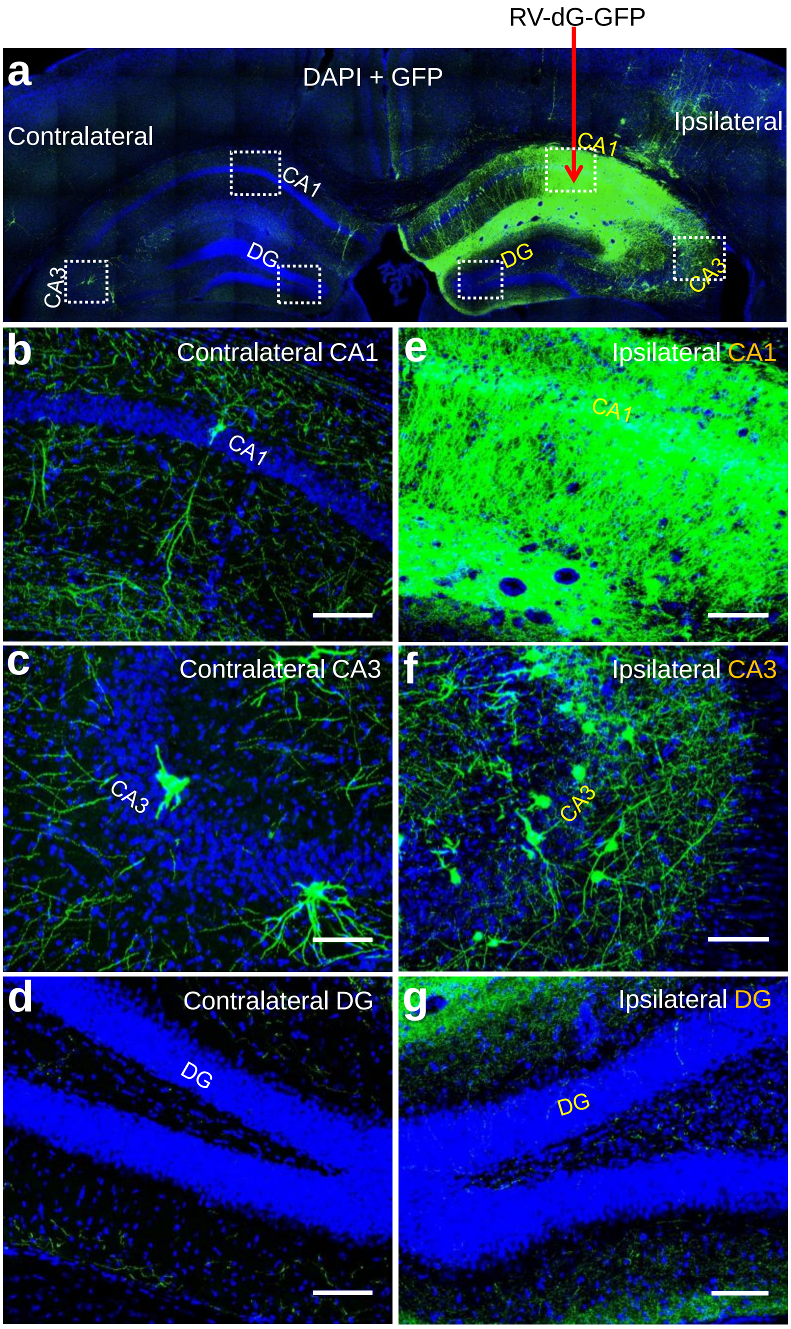

Supplementary Fig. 7 | The CA3-CA1 projections onto the CA1 stratum radium in the mouse. **a**, RV-dG–GFP virus (non-trans-synaptic tracing) was injected into in the stratum radiatum of ipsCA1. **b**, **e**, Almost no neurons was labelled in conCA1. **c**, **f**, Neurons were labelled in both ipsCA3 and conCA3, suggesting the CA3-CA1 projections. **d**, **g**, There was no neurons labelled in DG. ips, ipsilateral, con, contralateral, GFP, green, DAPI, blue. Calibration bar: 100 μm

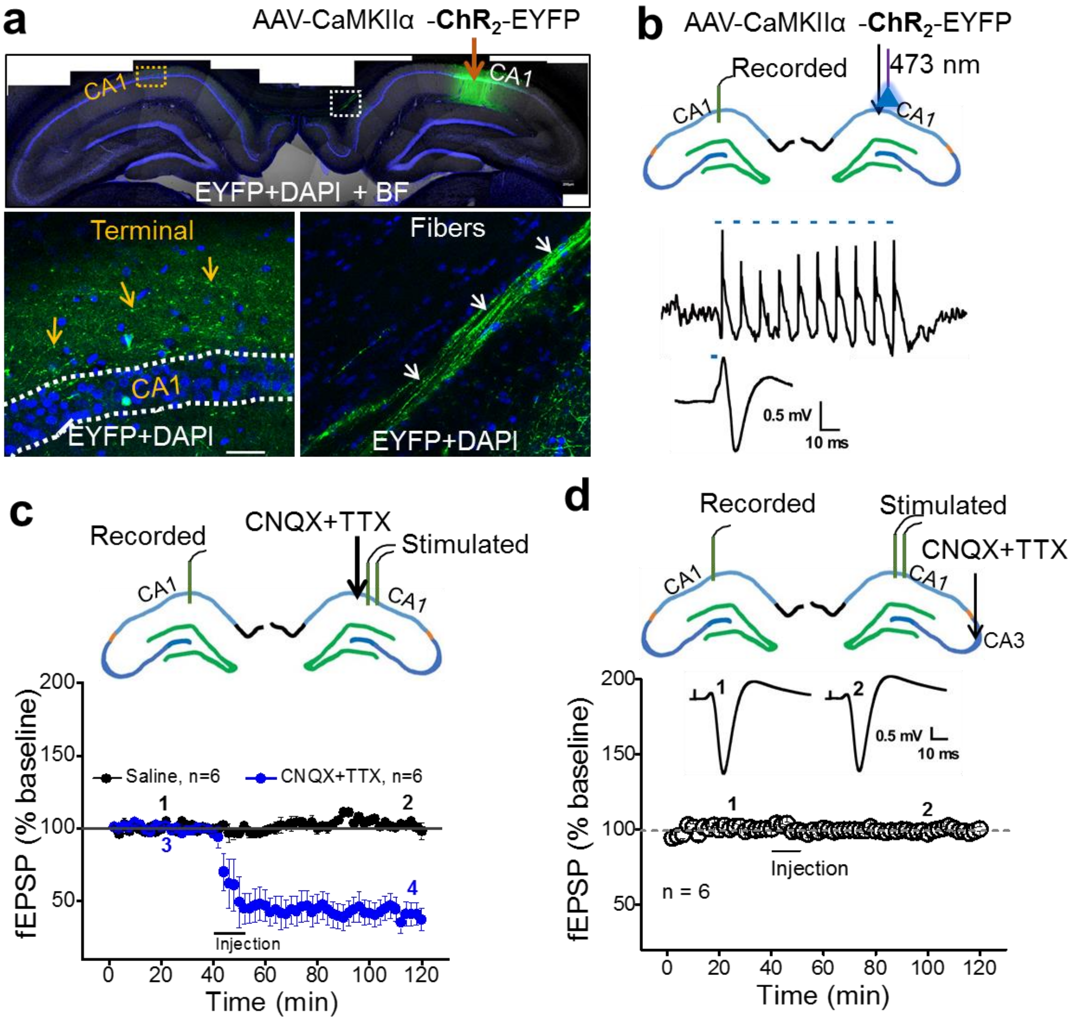

Supplementary Fig. 8 | Functional connectivity in the right CA1 projection onto the left CA1 stratum oriens. a, Right CA1 was injected with AAV-CaMKIIa-ChR2-EYFP and ample projection terminals were found in the stratum oriens of the opposite side. Calibration bar: 100 µm. **b**, Optogenetic stimulation at right CA1 evoked population spiking (middle panel) in the same side, while effectively evoked the fEPSP (lower panel) at the opposite side. **c**, The fEPSP recorded at left CA1 was largely reduced by infusion of CNQX+TTX into the stimulation side (right CA1) relative to saline treatment. **d**, In marked contrast, infusion of CNQX+TTX into right CA3 had no effects on the fEPSP recorded at left CA1. Error bars, s.e.m.

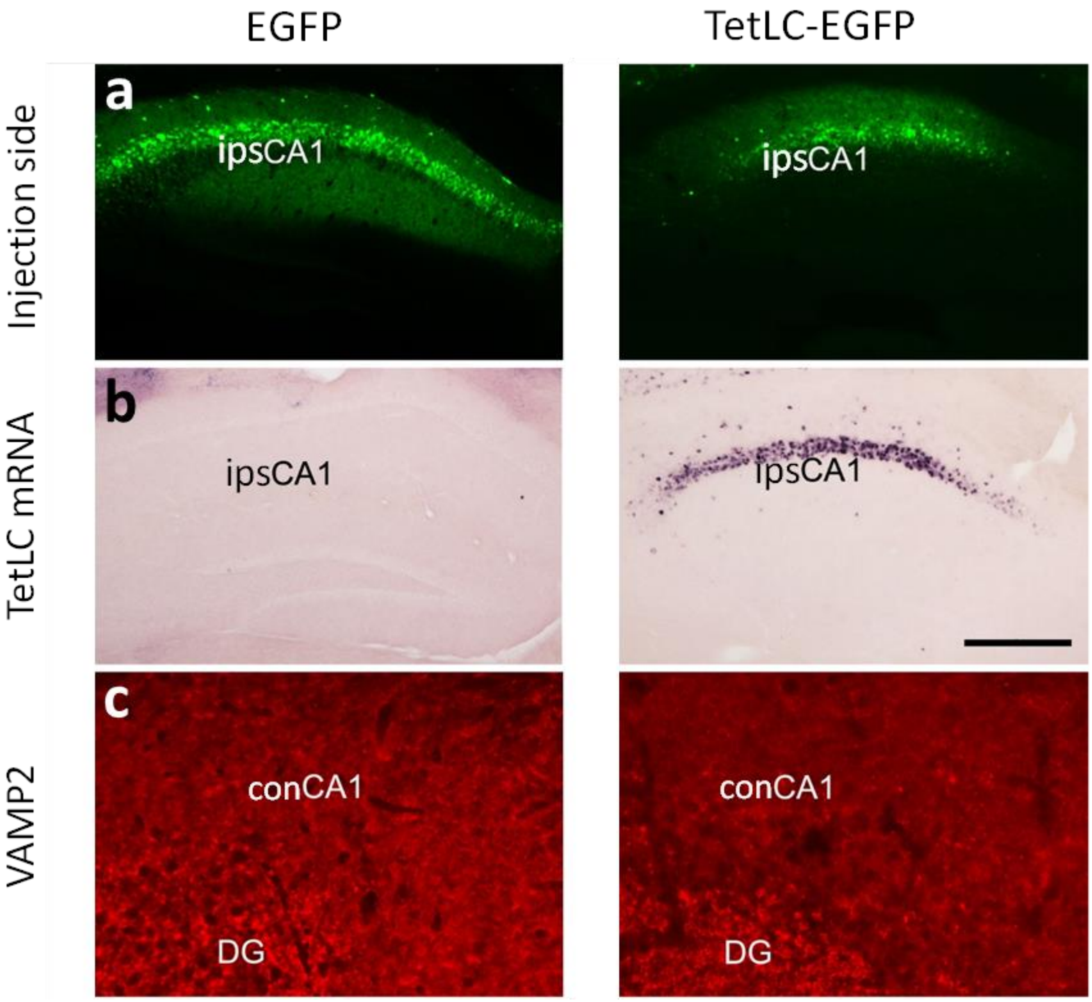

Supplementary Fig. 9 | VAMP2 expression in the opposite CA1 region to the injection of TetLC expressing virus. a, EGFP expression was observed in the ipsCA1 where AAV-syn-EGFP-2A-TetLC or control virus was injected. **b**, The TetLC expressing virus led to expression of TetLC mRNA (right) but not in control (left). **c**, The TetLC expressing virus but not control caused dramatically reduction of VAMP2 in the conCA1 to the virus injection side. ips, ipsilateral; con, contralateral. Calibration bar: 400 µm (**a**, **b**) and 100 µm (**c**).

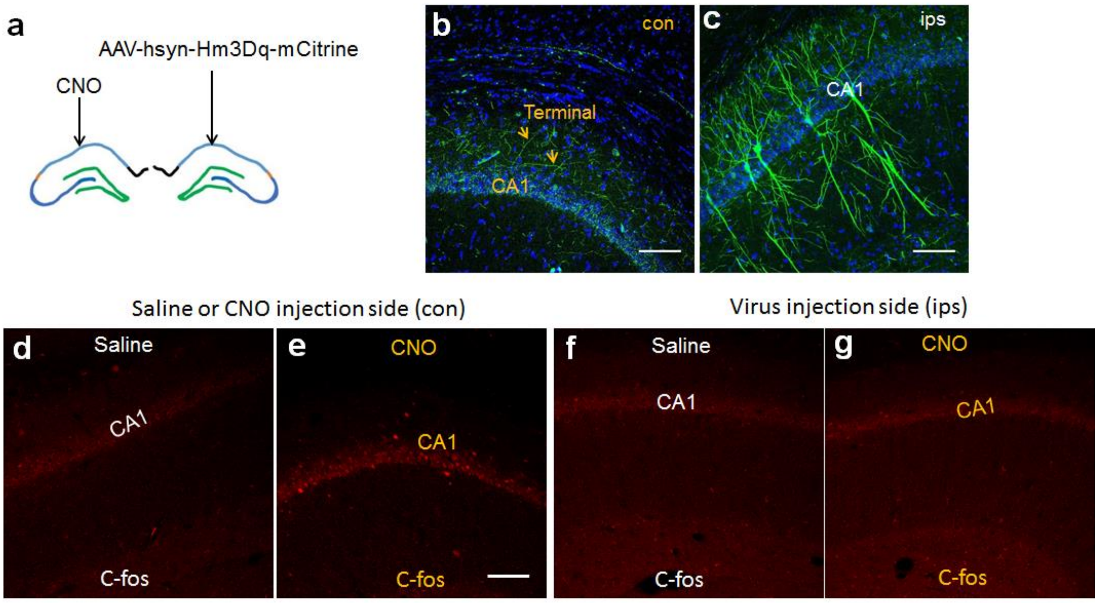

Supplementary Fig. 10 | Chemogenetic exciting of the ipsCA1-conCA1 synapses using hM3Dq. a, AAV-hsyn-hM3Dq-mCitrine was injected into ipsCA1 30 d before fear conditioning, and clozapine-N-oxide (CNO) was injected into conCA1 after fear conditioning. **b**, Ample terminals in the stratum oriens of conCA1 projected from ipsCA1 neurons (**c**). **d**, **e**, C-fos expression was enhanced with CNO but not saline injection in the conCA1. **f**, **g**, C-fos expression was not obviously affected in the other side where was injected with the Dq expressing virus. ips, ipsilateral; con, contralateral; mCitrine, green; DAPI, blue; C-fos, red. Calibration bar: 100 μm.

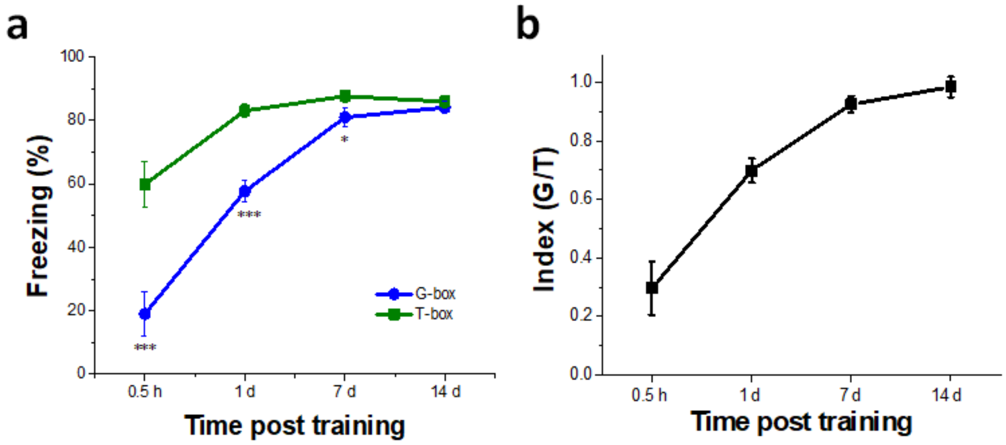

Supplementary Fig 11. | Slow generalisation of contextual fear memory in SD rat. On the acclimation day 24 h before contextual fear conditioning, rats were exposed to T-box but not G-box for 10 min. The other parts of the protocols were remained exactly the same as those described for rapid generalisation study. **a**, Independent groups of the rats (n = 10 for each group) were tested for contextual fear memory and generalisation at 0.5 h, and 1, 7 and 14 d after fear conditioning. The freezing level in G-box gradually risen to a near equivalent level to that in T-box on 14 d (Time×box interaction, F (3,36) = 16.557, *P* < 0.001; T-box vs. G-box contrast effects, ****P* < 0.001 at 0.5 h and 1 d; **P* = 0.026 on 7 d; *P* = 0.544 on 14 d; two-way ANOVA). **b**, The index calculated by the freezing level in G-box divided by that in T-box (G/T) indicated that generalisation development from about 30% to 98% within 14 d after fear conditioning, suggesting a time-course of slow generalisation in our experimental conditions over two weeks. This is in marked contrast to rapid generalisation that was fully developed within 24 h (see Fig. 1**b**). Error bars, s.e.m.
